# Aqueous Extract of *Phyllanthus niruri* Leaves Displays *In Vitro* Antioxidant Activity and Prevents the Elevation of Oxidative Stress in the Kidney of Streptozotocin-Induced Diabetic Male Rats

**DOI:** 10.1155/2014/834815

**Published:** 2014-06-01

**Authors:** Nelli Giribabu, Pasupuleti Visweswara Rao, Korla Praveen Kumar, Sekaran Muniandy, Somesula Swapna Rekha, Naguib Salleh

**Affiliations:** ^1^Department of Physiology, Faculty of Medicine, University of Malaya, 50603 Kuala Lumpur, Malaysia; ^2^Faculty of Agro Based Industry, University Malaysia Kelantan, Campus Jeli, Locked Bag No. 100, 17600 Jeli, Kelantan, Malaysia; ^3^Department of Biomedical Informatics, Division of Cancer Biology and System Biology, Asia University, Lioufeng Road, Wufeng, Taichung 41354, Taiwan; ^4^Department of Molecular Medicine, Faculty of Medicine, University of Malaya, Kuala Lumpur, Malaysia; ^5^Department of Zoology, Sri Venkateswara University, Tirupati, Andhra Pradesh 517502, India

## Abstract

*P. niruri* has been reported to possess antidiabetic and kidney protective effects. In the present study, the phytochemical constituents and *in vitro* antioxidant activity of *P. niruri* leaf aqueous extract were investigated together with its effect on oxidative stress and antioxidant enzymes levels in diabetic rat kidney. *Results*. Treatment of diabetic male rats with *P. niruri* leaf aqueous extract (200 and 400 mg/kg) for 28 consecutive days prevents the increase in the amount of lipid peroxidation (LPO) product, malondialdehyde (MDA), and the diminution of superoxide dismutase (SOD), catalase (CAT), and glutathione peroxidase (GPx) activity levels in the kidney of diabetic rats. The amount of LPO showed strong negative correlation with SOD, CAT, and GPx activity levels. *P. niruri* leaf aqueous extract exhibits *in vitro* antioxidant activity with IC_50_ slightly lower than ascorbic acid. Phytochemical screening of plant extract indicates the presence of polyphenols. *Conclusion*. *P. niruri* leaf extract protects the kidney from oxidative stress induced by diabetes.

## 1. Introduction

Oxidative stress results from an imbalance between radical-generating and radical-scavenging systems with increased production of reactive oxygen species (ROS) or reduced activity of antioxidant defences or both [1]. In oxidative stress, oxidation of macromolecules such as proteins, lipids, carbohydrates, and DNA is elevated. Hyperglycaemia has been identified as a major cause for ROS generation [2]. Hyperglycaemia causes glucose autooxidation, protein glycation, and advanced glycation end products (AGE) formation which could lead to the development of diabetic complications including retinopathy, neuropathy, and macro- and microvascular damage [3]. Oxidative stress has been considered as a common pathogenetic factor for diabetic nephropathy (DN), which is often associated with morphological changes of the kidney leading to end-stage renal failure (ESRF) [4, 5].

*Phyllanthus niruri* which belongs to the Euphorbiaceae family is also known as kidney stone crusher [6]. Traditionally,* P. niruri* is used to treat problems related to the gastrointestinal and genitourinary tracts [7]. There are reports which indicate that* P. niruri* can block calcium oxalate crystals [8] and stone formation in the kidney, ureter, and urinary bladder [9].* P. niruri* was also reported to display anticarcinogenic [10], hypolipidemic [11], hepatoprotective [12], anti-inflammatory [13], and antiplasmodial [14] effects as well as an effective treatment for hemorrhagic cystitis [15]. Recent studies have also revealed antidiabetic [16] and antioxidant properties of this herbal extract [9] both* in vivo* and* in vitro*.

In the present study, we hypothesized that* P. niruri* helps to prevent oxidative stress in the kidney of diabetics. Our study therefore investigated this plant effect on oxidative stress parameters, that is, activity levels of antioxidant enzymes (SOD, CAT, and GPx) and the amount of LPO product in the kidney of diabetic rats. In addition,* in vitro* antioxidant activity and phytochemical constituent of this plant extract were also investigated.

## 2. Material and Methods

### 2.1. Chemicals

DPPH (1,1-diphenyl,2-picrylhydrazyl), NBT (nitro blue tetrazolium), NADH (nicotinamide adenine dinucleotide phosphate reduced), PMS (phenazine methosulphate), ferrous chloride (FeCl_2_), streptozotocin, glibenclamide, epinephrine, thiobarbituric acid (TBA), reduced glutathione (GSH), and 5-5′-dithiobis(2-nitrobenzoic acid) (DTNB) were purchased from Sigma-Aldrich Co. (St Louis, Mo, USA). All other chemicals were of analytical grade.

### 2.2. Collection and Preparation of Plant Material

Fresh leaves of* P. niruri* were collected from Tirupati and authenticated by Dr. K. Madhava Chetty, Botanist, Sri Venkateswara University, Tirupati, India. The plant was deposited in Herbarium of Department of Botany, Sri Venkateswara University, Tirupati, India, with the number: 856201. The leaves of* P. niruri* were air-dried at room temperature and ground into powder. About 800 g of the powdered leaves was extracted in 1 L of cold sterile distilled water maintained on a mechanical shaker. The aqueous extract was filtrated with No. 1 Whatman Millipore filter paper (0.45 *μ*m Ref. HAWP04700, Bedford, MA, USA) and concentrated to dryness with a rotary evaporator (Rotavapor, R-210, Buchi Laborotechnik, AG, Flawil, Switzerland) at 50 ± 5C and lyophilized. A yielded freeze-dried material of approximately 36 g was obtained. The freeze-dried sample was stored in a cool dry place until ready for use.

### 2.3. Phytochemical Screening of* P. niruri* Leaves

The phytochemical components of* P. niruri* leaves were screened using standard method described by Harborne [17]. The leaf extract was screened for alkaloids, flavonoids, saponins, tannins, steroids, lignins, glycosides, terpenoids, polyphenols, coumarins, and resins. The colour intensity of the precipitate formed was used as analytical responses to these tests.

### 2.4. *In Vitro* Antioxidant Activities

Few different assays were performed to determine the antioxidative power of* P. niruri* leaf aqueous extract (20–300 *μ*g/mL) as described below. In each of these assays, ascorbic acid (20–300 *μ*g/mL) was used as a reference substrate. The ability of the extract to scavenge or inhibit free radicals was expressed as percentage inhibition and was calculated using the following formula:

(1) $$\begin{array}{c} \%\;\text{of}\;\text{inhibition}=\frac{\left(A_{0}-A_{t}\right)}{A_{0}}\times 100, \end{array}$$

where *A*_0_ is absorbance of the control group (without plant extract) and *A*_*t*_ is absorbance of* P. niruri* leaf extract. All determinations were carried out in triplicate.

#### 2.4.1. DPPH Radical Scavenging Activity

DPPH radical scavenging activity of* P. niruri* leaf aqueous extract was determined according to the method by Katalinic et al. [18]. In brief, 0.5 mL of 0.1 mM DPPH solution was prepared in methanol just before use. 1.0 mL of* P. niruri* leaf aqueous extract was added at different concentrations (20–300 *μ*g/mL) to DPPH solution. Double distilled H_2_O was used in the control group instead of samples, with the same procedures applied. The ability of the substrate to reduce the stable radical DPPH from deep purple to yellow-coloured diphenylpicrylhydrazine indicates its antioxidative potential. The mixture was shaken vigorously and left to stand for 30 min in the dark, and absorbance was measured at 517 nm using a spectrophotometer (UV-1700, Shimadzu, Kyoto, Japan). Lower absorbance at 517 nm represents higher DPPH scavenging activity.

#### 2.4.2. Superoxide Radical Scavenging Activity

Measurement of superoxide radical scavenging activity of* P. niruri* leaf extract followed the method by Xiang and Ning [19]. In brief, superoxide anions were generated in nonenzymatic phenazine methosulfate-nicotinamide adenine dinucleotide (PMS-NADH) system through the reaction of PMS, NADH, and oxygen. It was assayed by the reduction of NBT in the presence of different concentrations (20–300 *μ*g/mL) of the extract. The reaction was initiated by adding 0.75 mL of PMS (120 *μ*M) to the mixture. The absorbance was measured at 560 nm by using a spectrophotometer (UV-1700, Shimadzu, Kyoto, Japan) following 5-minute incubation at room temperature.

#### 2.4.3. Hydroxyl Radical Scavenging Activity

The hydroxyl radical scavenging activity of* P. niruri* leaf extract was measured according to a modified method by Eswar Kumar et al. [20]. The reaction mixture contained 60 *μ*L of 1.0 mM FeCl_2_, 90 *μ*L of 1 mM 1,10-phenanthroline, 2.4 mL of 0.2 M phosphate buffer (pH 7.8), 150 *μ*L of 0.17 M hydrogen peroxide (H_2_O_2_), and 1.5 mL of different concentrations of the extract (20–300 *μ*g/mL). H_2_O_2_ was added at the start of the reaction. After incubation at room temperature for 5 min, absorbance of the mixture was measured by using a spectrophotometer (UV-1700, Shimadzu, Kyoto, Japan) at 560 nm.

#### 2.4.4. Hydrogen Peroxide Scavenging Activity

The ability of* P. niruri* leaf extract to scavenge hydrogen peroxide was determined according to the method by Ruch et al. [21]. A solution of hydrogen peroxide (40 mM) was prepared in phosphate buffer (pH 7.4).* P. niruri* leaf extract at different concentrations (20–300 *μ*g/mL) was added to hydrogen peroxide solution (0.6 mL and 40 mM). Absorbance of the mixture was determined after 10 minutes against a blank solution containing phosphate buffer by using a spectrophotometer (UV-1700, Shimadzu, Kyoto, Japan) at 230 nm.

### 2.5. Experimental Animals

Adult male Wistar rats weighing 180–210 g were obtained from Animal House, Faculty of Medicine, University of Malaya, Kuala Lumpur, Malaysia. Five to six animals were housed together under a standard environmental condition of temperature 25 ± 2°C, relative humidity between 45 and 55%, and 12 hr light/dark cycle. Rats had free access to standard food pellet (Harlan diet, UK) and water* ad libitum*. The experimental protocol was in accordance with ARRIVE guidelines (Animals in Research: Reporting* In Vivo* Experiments) and European Community Guidelines (EEC Directive, 1986). This study was approved by the Faculty of Medicine, Animal Care and Use Committee, University of Malaya, with ethics number: 2013-07-15/FIS/R/NS. Acute toxicity study was conducted according to the Organization for Economic Cooperation and Development (OECD) revised up-and-down procedure for acute toxicity testing (OECD guideline 425) [22]. No signs of acute toxicity were observed in the tested animals.

### 2.6. Induction of Diabetes

Diabetes mellitus was artificially induced in overnight-fasted rats by a single intraperitoneal injection of STZ (55 mg/kg bw) dissolved in 0.1 M cold citrate buffer (pH 4.5) [23]. Rats were allowed to drink 5% glucose solution overnight to overcome drug-induced hypoglycemia. STZ was reported to selectively destroy the pancreatic *β* cells [23]. Diabetes was confirmed by the presence of polyuria, polydipsia, and weight loss and only animals exhibiting blood glucose levels above 300 mg/dL on the third day following STZ injection were considered as diabetics. Treatment was started three days after STZ injection which was considered to be day one.* P. niruri* leaf aqueous extract was administered orally by using a gavage tube daily for 28 consecutive days. The extract was administered in the form of suspension in 1% sodium carboxymethyl cellulose (Na-CMC) dissolved in distilled water.

### 2.7. Experimental Design

Animals were randomly divided into five groups with six rats in each group:

 Group 1: normal and nondiabetic: received 1% Na-CMC vehicle only;
 Group 2: diabetic: received 1% Na-CMC vehicle only;
 Groups 3 and 4: diabetic: treated with* P. niruri* leaf aqueous extract at 200 mg/kg [24] and 400 mg/kg [25] body weight, respectively;
 Group 5: diabetic: treated with standard antidiabetic agent, glibenclamide at 600 *μ*g/kg body weight.

### 2.8. Collection of Tissue Samples

After 28-day treatment, rats were fasted overnight prior to sacrifice. Intraperitoneal injection of pentobarbitone sodium anesthesia (60 mg/kg) was given prior to cervical dislocation. Kidneys were immediately harvested and washed with ice-cold saline, immersed in liquid nitrogen, and stored at −80°C for biochemical analysis.

### 2.9. Preparation of Kidney Cytosolic Fraction

Kidney was weighed and 10% of tissue homogenate was prepared in phosphate buffer (0.1 m, pH 7.4) using a glass-Teflon homogenizer (Heidolph Silent Crusher M, Germany). Homogenates were then centrifuged for 10 min, 500 g at 4°C. Supernatant was collected and recentrifuged at 2000 g for 10 min. Supernatant was again collected and recentrifuged at 12000 g for 10 min at 4°C and pellet was resuspended in 200 mM mannitol, 50 mM sucrose, and 10 mmol/L Hepes-KOH (pH 7.4). The final supernatant was taken and centrifuged for 1 h at 40000 g [26]. The cytosolic fraction was frozen at −80°C until further used.

### 2.10. Estimation of LPO Product

LPO was estimated by thiobarbituric acid (TBA) reaction with malondialdehyde (MDA), where the latter was a product formed from membrane lipid peroxidation [27]. In brief, 2.5 mL homogenate, 0.5 mL of 0.9% NaCl, and 1.0 mL 20% w/v TCA were added into the mixture. The mixture was then centrifuged for 20 minutes at 4000 ×g at 4°C. 0.25 mL TBA reagent was added to 1.0 mL supernatant and the mixture was incubated at 95°C for 1 hr and cooled under running tap water prior to addition of 1 mL *n*-butanol. After a thorough mixing, the mixture was centrifuged for 15 minutes at 4000 ×g at 4°C. The organic layer was transferred into a clear tube and absorbance was measured at 532 nm with a spectrophotometer (UV-1700, Shimadzu, Kyoto, Japan). The rate of lipid peroxidation was expressed as *μ* moles of MDA formed/gram wet weight of the tissue.

### 2.11. Estimation of SOD Activity

SOD activity was assayed according to the method by Misra and Fridovich [28]. The assay procedure involves inhibition of epinephrine autooxidation to adrenochrome in an alkaline medium (pH 10.2), which was markedly inhibited in the presence of SOD. 1.5 mL carbonate buffer (0.05 M, pH 10.2) and 0.5 mL ethylenediaminetetraacetic acid (EDTA) (0.49 M) were added to 0.5 mL supernatant. The reaction was initiated by the addition of 0.4 mL epinephrine (3 mM). Changes in absorbance were recorded at 480 nm for one min at 15 sec interval, 3 min each, by using a spectrophotometer (UV-1700, Shimadzu, Kyoto, Japan). SOD activity levels were expressed as the amount of enzyme that inhibits oxidation of epinephrine by 50%, which was equal to 1 U per milligram of protein.

### 2.12. Estimation of CAT Activity

CAT enzyme activity was determined on the basis of hydrogen peroxide decomposition [29]. The reaction solution contained 2.5 mL of 50 mmol phosphate buffer (pH 5.0) and 0.4 mL of 5.9 mmol H_2_O_2_. The reaction was initiated by adding 0.1 mL enzyme extract. Changes in the absorbance of the reaction solution were monitored every 30*s* and was read by using a spectrophotometer (UV-1700, Shimadzu, Kyoto, Japan) at 240 nm. The enzyme activity levels were expressed as *μ*mol of hydrogen peroxide (H_2_O_2_) metabolized/mg protein/min.

### 2.13. Estimation of GPx Activity

GPx activity was measured according to the method by Rotruck et al. [30]. The reaction mixture consists of 0.2 mL, 0.8 mM EDTA; 0.1 mL, 10 mM sodium azide; 0.1 mL, 2.5 mM H_2_O_2_; 0.2 mL GSH; 0.4 mL, 0.4 mM phosphate buffer (pH 7.0); and 0.2 mL homogenate which were incubated at 37°C for 10 minutes. The reaction was arrested by the addition of 0.5 mL of 10% TCA and was centrifuged at 2000 rpm. 3.0 mL of 0.3 M disodium hydrogen phosphate and 1.0 mL of DTNB were added to the supernatant and the colour changes were read immediately by using a spectrophotometer (UV-1700, Shimadzu, Kyoto, Japan) at 420 nm. GPx activity levels were expressed as *μ*Mol of GSH consumed/mg protein/min.

### 2.14. Statistical Analysis

Statistical differences were evaluated by analysis of variance (ANOVA) followed by Student's* t*-test. *P* value < 0.05 was considered to be significant. Post hoc statistical power analysis was performed and all values obtained were >0.8 which indicate adequate sample size. Shapiro-Wilk test results were >0.05 which indicate data normality.

## 3. Results

### 3.1. Phytochemical Screening

Preliminary phytochemical screening showed the presence of alkaloids, flavonoids, saponins, tannins, lignins, terpenoids, polyphenols, and coumarins; however, steroids, glycosides, and resins were not detected in the leaf extract of* P. niruri* (data not shown).

### 3.2. *In Vitro* Antioxidant Activities

#### 3.2.1. DPPH Radical Scavenging Activity

**Figure 1:**
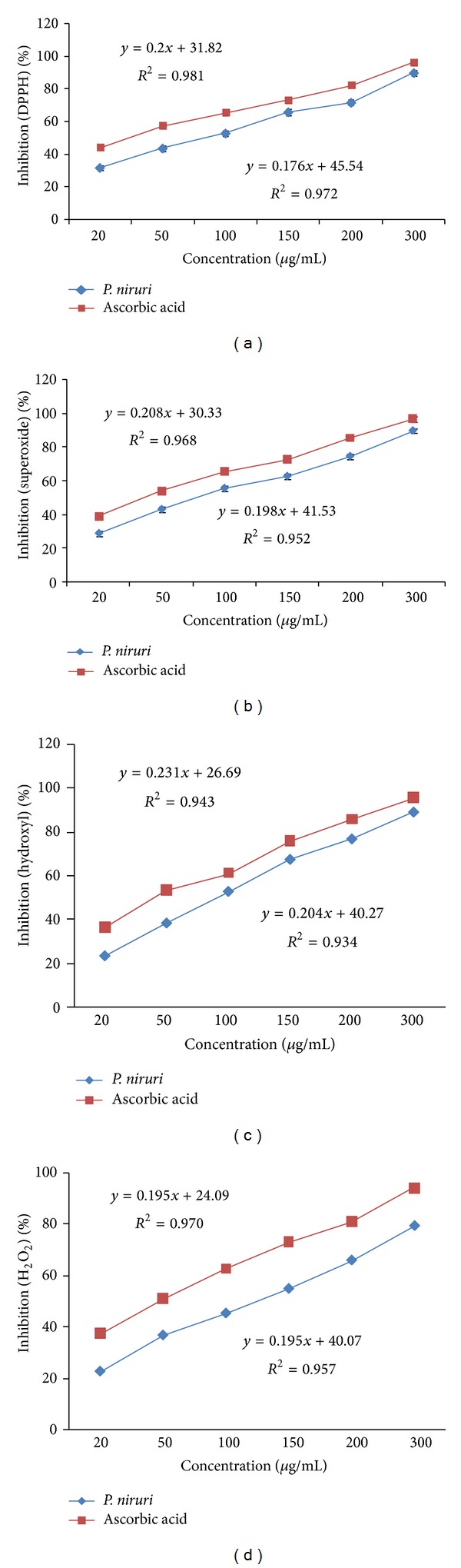
*In vitro* antioxidant assay of* P. niruri* leaf aqueous extract. The graphs show (a) DPPH radical, (b) superoxide radical, (c) hydroxyl radical, and (d) hydrogen peroxide scavenging activities of* P. niruri* leaf extract and ascorbic acid. Results represent means of triplicates of each concentration. For each assay, the IC_50_ for* P. niruri* leaf extract was slightly less than ascorbic acid.

DPPH radical scavenging activity of aqueous leaf extract of* P. niruri* is presented in Figure 1(a). Our findings indicate that 20 *μ*g/mL of the leaf extract and ascorbic acid conferred 31.66% and 43.89% inhibition on DPPH radicals, respectively. The IC_50_ for* P. niruri* leaf extract and ascorbic acid was 90.86 *μ*g/mL and 25.31 *μ*g/mL, respectively. The IC_50_ of the leaf aqueous extract was 3.58 times lower than ascorbic acid. The extract exhibits gradual dose-dependent increase on DPPH inhibition.

#### 3.2.2. Hydroxyl Radical Scavenging Activity

Hydroxyl radical scavenging activity of aqueous leaf extract of* P. niruri* is presented in Figure 1(b). At 20 *μ*g/mL, the aqueous leaf extract and ascorbic acid inhibit hydroxyl radical by 23.66% and 36.56%, respectively.* P. niruri* leaf aqueous extract displays a dose-dependent inhibition on hydroxyl radicals with IC_50_ of 100.6 *μ*g/mL. The IC_50_ for ascorbic acid was 47.44 *μ*g/mL. The IC_50_ of* P. niruri* leaf was 2.12 times lower than ascorbic acid.

#### 3.2.3. Superoxide Radical Scavenging Activity

Figure 1(c) shows dose-dependent scavenging activity of* P. niruri* leaf aqueous extract and ascorbic acid on superoxide radical. At 20 *μ*g/mL, the percentage inhibition of* P. niruri* leaf aqueous extract and ascorbic acid was 28.46% and 38.67%, respectively. The IC_50_ for* P. niruri* leaf extract was 94.34 *μ*g/mL, while ascorbic acid was 42.64 *μ*g/mL. The IC_50_ of* P. niruri* leaf extract was 2.21-fold lower than ascorbic acid.

#### 3.2.4. Hydrogen Peroxide Scavenging Activity

The ability of* P. niruri* leaf aqueous extract and ascorbic acid to scavenge hydrogen peroxide free radical is shown in Figure 1(d). H_2_O_2_ inhibition by* P. niruri* leaf extract and ascorbic acid at 20 *μ*g/mL was 22.54% and 37.57%, respectively. The IC_50_ for* P. niruri* leaf extract and ascorbic acid was 132.57 *μ*g/mL and 50.81 *μ*g/mL, respectively. The IC_50_ of* P. niruri* leaf aqueous extract was 2.61-fold lower than ascorbic acid.

### 3.3. Effect of* P. niruri* Leaf Aqueous Extract on Renal MDA Levels

**Figure 2:**
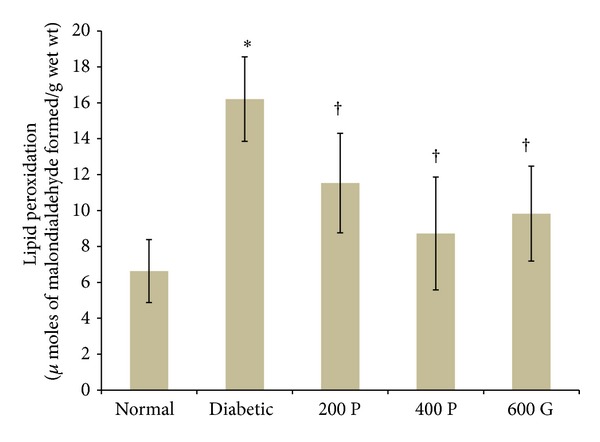
Estimation of LPO product, MDA, in the kidney in different experimental groups. Higher MDA levels were noted in diabetic rats as compared to normal, nondiabetic control rats. Administration of* P. niruri* leaf extract resulted in decreased MDA levels in the kidney in diabetes. 200P: 200 mg/kg/day* P. niruri *leaf extract; 400P: 400 mg/kg/day* P. niruri* leaf extract; 600G: 600 *μ*g/kg/day glibenclamide. *n* = 6 per treatment group, *P < 0.05 as compared to normal, nondiabetic control rats, and ^†^ *P* < 0.05 as compared to nontreated diabetic rats.

The levels of renal LPO product, MDA, were significantly higher in nontreated diabetic rats (144.49%) as compared to normal, nondiabetic rats (Figure 2). Administration of 200 mg/kg* P. niruri* leaf extract to diabetic rats caused the levels of MDA to decrease by 28.87% as compared to the nontreated diabetic rats. Meanwhile, the levels of MDA were 46.2% lower in diabetic rats treated with 400 mg/kg* P. niruri* leaf aqueous extract as compared to the nontreated diabetic rats (Figure 2). In glibenclamide-treated diabetic rats, the levels of MDA were 39.35% lower than the nontreated diabetic rats. 400 mg/kg* P. niruni* leaf extract was 0.88-fold less potent than glibenclamide in preventing the increase in LPO product in the kidney of diabetic rats (Figure 2).

### 3.4. Effect of* P. niruri* Leaf Aqueous Extract on Renal SOD Levels

**Figure 3:**
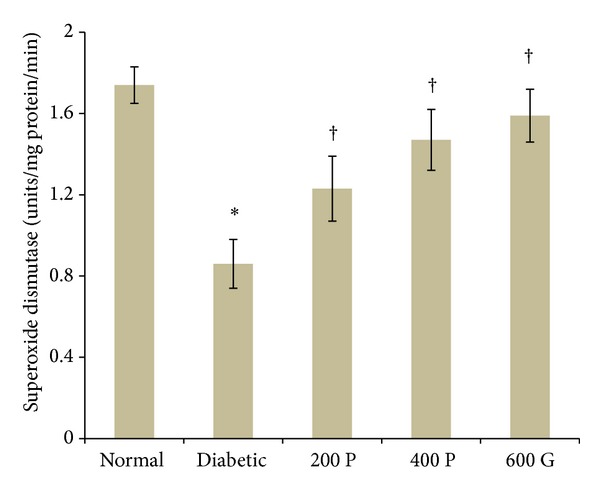
SOD activity levels in the kidney in different experimental groups. SOD activity levels were reduced in diabetic rats as compared to normal, nondiabetic rats. Administration of* P. niruri* leaf extract at 200 or 400 mg/kg/day and glibenclamide resulted in higher SOD activity in the kidney as compared to nontreated diabetic rats. 200P: 200 mg/kg/day* P. niruri* leaf extract; 400P: 400 mg/kg/day* P. niruri* leaf extract; 600G: 600 *μ*g/kg/day glibenclamide. *n* = 6 per treatment group, *P < 0.05 as compared to normal, nondiabetic control rats, and ^†^ *P* < 0.05 as compared to nontreated diabetic rats.

Figure 3 shows the effect of* P. niruri* leaf aqueous extract and glibenclamide on SOD activity levels in diabetic rat kidney. In the nontreated diabetic rats, SOD activity level was 50.57% lower than normal, nondiabetic rats. However, renal SOD activity level was 43.02% higher in diabetic rats treated with 200 mg/kg* P. niruri* leaf extract as compared to the nontreated diabetic rats. Following treatment with 400 mg/kg *P. niruri* leaf extract, the SOD activity levels were 79.93% higher than the nontreated diabetic rats. Glibenclamide-treated diabetic rats have 84.88% higher renal SOD activity level as compared to the nontreated diabetic rats. Treatment with 400 mg/kg* P. niruri* leaf aqueous extract was less potent than glibenclamide in preventing the decrease in SOD activity levels in diabetic rat kidney.

### 3.5. Effect of* P. niruri* Leaf Aqueous Extract on Renal CAT Levels

**Figure 4:**
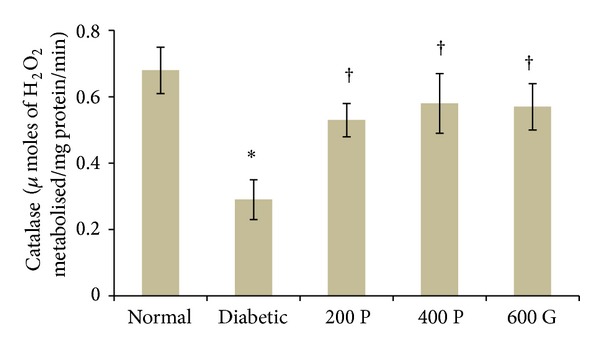
CAT activity levels in the kidney in different experimental groups. Administration of* P. niruri* leaf extract at 200 and 400 mg/kg/day or glibenclamide to diabetic rats prevented decrease in CAT activity in the kidney in diabetes. 200P: 200 mg/kg/day* P. niruri* leaf extract; 400P: 400 mg/kg/day* P. niruri* leaf extract; 600G: 600 *μ*g/kg/day glibenclamide. *n* = 6 per treatment group, *P < 0.05 as compared to normal, nondiabetic control rats, and ^†^ *P* < 0.05 as compared to nontreated diabetic rats.

The activity levels of CAT in the kidney of normal, STZ-induced diabetic rats and diabetic rats which received* P. niruri* leaf aqueous extract or glibenclamide are presented in Figure 4. Our findings indicate that CAT activity levels were 57.35% lower in STZ-induced diabetic rat kidney as compared to normal, nondiabetic rats. 28-day treatment with 200 mg/kg/day* P. niruri* leaf aqueous extract resulted in higher CAT activity level (82.75%) as compared to the nontreated diabetic rats. Treatment with 400 mg/kg/day* P. niruri* leaf aqueous extract resulted in 100% higher CAT activity levels as compared to the nontreated diabetic rats. Meanwhile, glibenclamide treatment resulted in 96.55% higher CAT activity levels as compared to the nontreated diabetic rats. No significant difference in CAT activity levels was noted between treatment with 400 mg/kg/day* P. niruri* leaf extract and glibenclamide.

### 3.6. Effect of* P. niruri* Leaf Aqueous Extract on Renal GPx Levels

**Figure 5:**
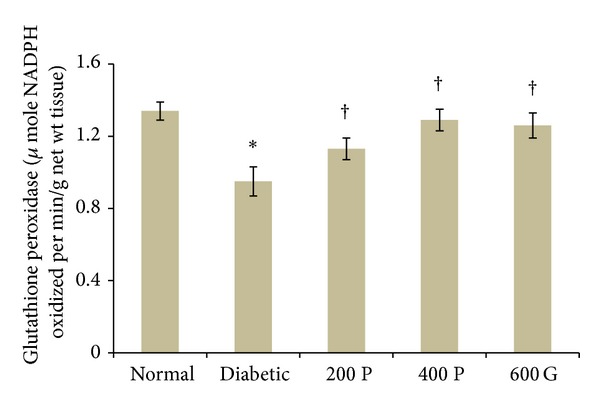
GPx activity levels in the kidney in different experimental groups. Administration of* P. niruri* leaf extract at 200 and 400 mg/kg/day or glibenclamide prevented deterioration of GPx activity levels in the kidney in diabetes. 200P: 200 mg/kg/day* P. niruri* leaf extract; 400P: 400 mg/kg/day* P. niruri* leaf extract; 600G: 600 *μ*g/kg/day glibenclamide. *n* = 6 per treatment group, *P < 0.05 as compared to normal, nondiabetic control rats, and ^†^ *P* < 0.05 as compared to nontreated diabetic rats.

Figure 5 shows the effect of* P. niruri* leaf aqueous extract or glibenclamide on renal GPx levels in diabetic rats. Our findings indicate that GPx activity levels were lower in diabetic rats (29.1%) as compared to normal, nondiabetic rats. Treatment with 200 mg/kg/day and 400 mg/kg/day aqueous leaf extract of* P. niruri* resulted in a significantly higher GPx activity levels (18.94% and 35.78%, resp.) as compared to the nontreated diabetic rats. Glibenclamide treatment resulted in 32.63% higher GPx activity levels in diabetic rats as compared to the nontreated diabetic rats. No significant difference in GPx activity levels was noted between treatment with* P. niruri* leaf aqueous extract and glibenclamide.

### 3.7. Correlation between Levels of MDA and Antioxidant Enzymes in the Kidney

**Figure 6:**
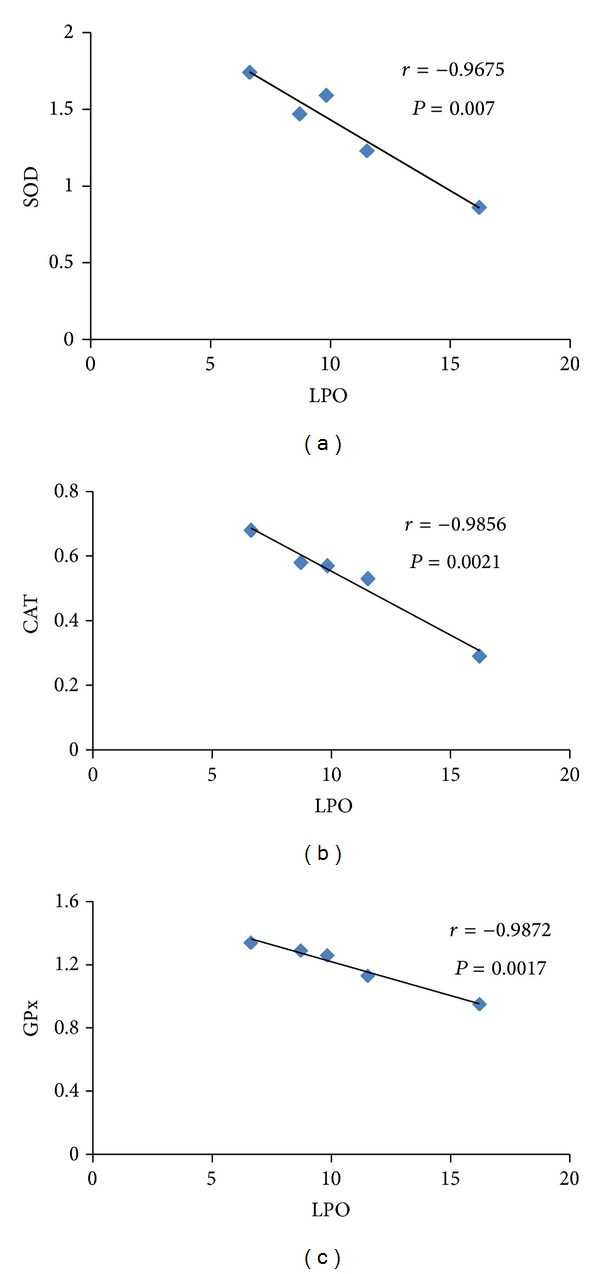
(a–c) Correlation between MDA and activity levels of SOD, CAT, and GPx in the kidney. Strong negative correlations were noted between the levels of MDA and activity of antioxidant enzymes indicating that the extent of tissue damage due to oxidative stress was directly related to the levels of endogenous antioxidant enzymes.

Negative correlations were observed between the levels of kidney LPO products as reflected by MDA amount (Figures 6(a), 6(b), and 6(c)) and SOD, CAT, and GPx activity levels. A strong negative correlation was observed between renal MDA content and SOD activity levels (*r* = −0.9675, *P* = 0.007), MDA content and CAT activity levels (*r* = −0.9856, *P* = 0.0021), and renal MDA content and GPx activity levels (Figure 6(c)), with correlation coefficient = −0.9872,*P* = 0.0017.

## 4. Discussion

Hyperglycaemia increases the production of free radicals and decreases the tissue antioxidative capacity in diabetes. These imbalances lead to tissue oxidative stress. The tissue antioxidative potential needs to be raised in order to overcome oxidative damage. The kidney is involved in maintaining body homeostasis as well as regulating electrolytes and acid-based balances and blood pressure. Diabetes-related kidney oxidative stress was reported to cause glomerular hypertrophy, basement membrane thickening, mesangial expansion, tubular atrophy, interstitial fibrosis, and arteriolar thickening [31]. A number of medicinal plants have been reported to possess antioxidative capacity which could help reduce free radical formation and promote endogenous antioxidant enzyme activity in the kidney [18]. One of the plants which has been reported to display kidney protective effect is* P. niruri*. The aqueous extract of* P. niruri* has been shown to reduce the crystals aggregation in rat urine which could help to inhibit the formation of urinary stones [8]. In the present study, aqueous leaf extract of* P. niruri* was found to display* in vitro* free radical scavenging activity, reduce endogenous LPO product formation, and increase activity levels of endogenous antioxidant enzymes in the kidney, which makes this herb a potential treatment for diabetic nephropathy.

We have shown that the aqueous leaf extract of* P. niruri* displays* in vitro* antioxidant activity from the dose-dependent inhibition on DPPH, superoxide and hydroxyl radicals formation. Additionally, this extract also possesses hydrogen peroxide scavenging activity. Together with superoxide inhibition,* P. niruri* leaf aqueous extract could inhibit formation of peroxides and breaks autooxidative chain reaction. Our findings were consistent with the previous reports on* in vitro* radical scavenging activity of* P. niruri* [32, 33]. The free radical scavenging activity of this herb could be due to the presence of various bioactive compounds such as alkaloids, flavonoids, coumarins, and polyphenols which were reported to possess antioxidant capabilities [34].

Many studies have shown that hyperglycaemia was able to increase tissue oxidative stress via activating the polyol pathway, nonenzymatic protein glycosylation, and autooxidation of glucose leading to increased production of reactive oxygen species (ROS) which include superoxide radical (O^2•−^), hydrogen peroxide (H_2_O_2_), and hydroxyl radical (OH^•^). Additionally, hyperglycemia could also reduce antioxidant defence systems of the body [35, 36]. Experimentally induced diabetic rats were known to have high levels of tissue oxidative stress as characterized by high amount of LPO products, MDA, which indirectly reflects intensified free radical production [37]. The end product of lipid peroxidation was found to cause damage to the proteins, lipids, and DNAs [38]. In the present study, MDA levels in diabetic rat kidneys were higher than nondiabetic rats. Recently, Naik et al. [39] and others [40, 41] have reported that tissue oxidative damage as reflected by high amount of lipid peroxidation products occurs in the kidney of diabetic rats. Our findings which indicate a significantly lower LPO product accumulation in the kidney of diabetic rats following* P. niruri* leaf aqueous extract treatment provide evidence that consumption of this extract could prevent elevation of oxidative stress in the kidney in diabetes. Other* Phyllanthus* species such as* Phyllanthus amarus* have been reported to protect the kidney against the increase in LPO product in diabetes [42].

The physiological levels of antioxidant enzymes such as SOD, CAT, and GPx are important to reduce the formation of H_2_O_2_ by dismutating oxygen radicals, eliminating organic peroxides, and reducing hydroperoxides generations in hyperglycaemic condition [43]. SOD is involved in scavenging superoxide radicals (O^2•−^) and therefore prevents its conversion into H_2_O_2_ and molecular oxygen [35]. In our study, SOD activity levels were reduced in diabetic rat kidney which could be due to oxidative inactivation by H_2_O_2_ or glycosylation [44]. Fujita et al. [45] reported that downregulation of renal CuZn-SOD (SOD1) and CuZn-SOD (SOD3) activities could result in diabetic nephropathy. Kitada et al. [46] reported that resveratrol, a plant bioactive compound, prevents nephropathy development by preventing Mn-SOD dysfunction in the kidney of diabetic mice. In view of this, the effect of the leaf extract of* P. niruri* in maintaining renal SOD activity levels near normal could help to preserve the kidney function and prevent nephropathy development in diabetes. Our previous study has shown that the aqueous leaf extract of* P. niruri* prevents the decrease in SOD activity in cardiac tissue following doxorubicin-induced myocardial toxicity in rats [47].

CAT and GPx are involved in the elimination of H_2_O_2_ [48]. CAT was regarded as a major renal antioxidant which helps to reduce H_2_O_2_ and protects the tissues from highly reactive hydroxyl radicals [49]. In our study, decreased CAT activity in the kidney was observed in diabetic rats. Hwang et al. [50] reported that CAT deficiency could accelerate kidney injury in diabetes through peroxisomal dysfunction. Administration of* P. niruri* leaf aqueous extract to diabetic rats could prevent the decrease in renal CAT activity most probably via preventing dysfunction of CAT enzyme by free radicals. GPx, a selenium containing enzyme, plays a role in minimizing tissue oxidative damage [41]. Reduced GPx activity in diabetes could be due to inactivation by free radicals [51]. In our study, GPx activity levels were maintained near normal in diabetic rat kidneys following* P. niruri* leaf aqueous extract treatment which suggest that this herb protects this tissue against oxidative damage by preventing enzyme dysfunction. A strong negative correlation between LPO and SOD and CAT and GPx activity levels indicated that kidney oxidative damage was dependent on activity levels of these enzymes. Additionally, the decreased levels of LPO product following* P. niruri* leaf aqueous extract treatment could also be due to reduced level of free radicals as observed from* in vitro* radical scavenging effect of the leaf extract.

In conclusion, this study has provided scientific evidence, whereby administration of* P. niruri* leaf aqueous extract to diabetic rats could reduce oxidative stress in the kidney via preventing the decrease in activity levels of endogenous antioxidant enzymes. Our findings therefore support the claims that this herb is beneficial in treating kidney disease due to diabetes.
